# Structure-based development of novel sirtuin inhibitors

**DOI:** 10.18632/aging.100388

**Published:** 2011-09-20

**Authors:** Christine Schlicker, Gina Boanca, Mahadevan Lakshminarasimhan, Clemens Steegborn

**Affiliations:** ^1^ Department of Physiological Chemistry, Ruhr-University Bochum, Germany; ^2^ Present address: Department of Biophysics, Ruhr-University Bochum, Germany; ^3^ Department of Biochemistry, University of Bayreuth, Germany

**Keywords:** Sirtuins, docking, Sirt2, inhibitors, isoform-specific, structure-based drug development, aging-related diseases

## Abstract

Sirtuins are NAD^+^-dependent protein deacetylases regulating metabolism, stress responses, and aging processes. Mammalia possess seven Sirtuin isoforms, Sirt1-7, which differ in their subcellular localization and in the substrate proteins they deacetylate. The physiological roles of Sirtuins and their potential use as therapeutic targets for metabolic and aging-related diseases have spurred interest in the development of small-molecule Sirtuin modulators. Here, we describe an approach exploiting the structures available for four human Sirtuins for the development of isoform-specific inhibitors. Virtual docking of a compound library into the peptide binding pockets of crystal structures of Sirt2, 3, 5 and 6 yielded compounds potentially discriminating between these isoforms. Further characterization in activity assays revealed several inhibitory compounds with little isoform specificity, but also two compounds with micromolar potency and high specificity for Sirt2. Structure comparison and the predicted, shared binding mode of the Sirt2-specific compounds indicate a pocket extending from the peptide-binding groove as target side enabling isoform specificity. Our family-wide structure-based approach thus identified potent, Sirt2-specific inhibitors as well as lead structures and a target site for the development of compounds specific for other Sirtuin isoform, constituting an important step toward the identification of a complete panel of isoform-specific Sirtuin inhibitors.

## INTRODUCTION

Sirtuin proteins are protein deacetylases that contribute to the regulation of metabolism, stress responses, and aging processes [1-3]. They form class III of the protein deacetylase superfamily and hydrolyze one nico-tinamide adenine dinucleotide (NAD^+^) cosubstrate for each protein lysine side chain they deacetylate [4]. The seven mammalian Sirtuins (Sirt1-7) show different intracellular localization [5] and deacetylate different sets of substrate proteins. Sirt1 locates to the nucleus and regulates, e.g., transcription factors such as p53 and PGC-1α[1, 6]. Sirt6 and Sirt7 are also nuclear isoforms; Sirt7 regulates RNA polymerase I [7] and can deacetylate p53 [8], whereas Sirt6 deacetylates histones and regulates DNA stability and repair [9-11]. Sirt2 mainly resides in the cytosol where it can deacetylate α-tubulin [12]. Sirt3, 4, and 5 are located in mitochondria[5,13]. Sirt3 appears to regulate a large set of metabolic enzymes, whereas only one physiological Sirt5 substrate is known, carbamoylphosphate synthetase I [13-19]. Sirt4 is the only mammalian Sirtuin without known deacetylation substrate. Instead, Sirt4 was shown to ADP-ribosylate - a second type of reaction that can be catalyzed by Sirtuins - glutamate dehydrogenase [20].

**Figure 1:**
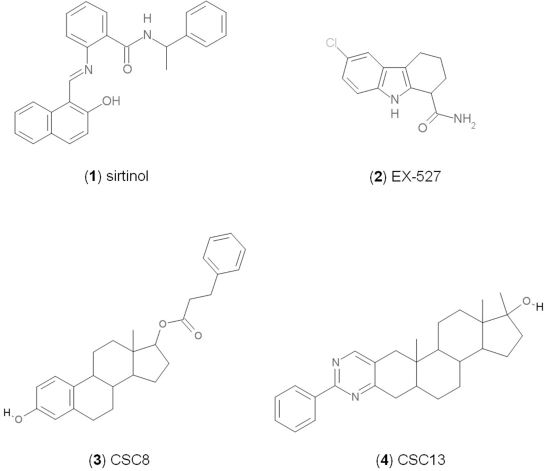
Chemical structures of known and novel Sirtuin inhibitors Sirtinol (**1**) and EX-527 (**2**) are known Sirtuin inhibitors. **1** shows low potency and limited discrimination between Sirt1 and Sirt2. 2 is a potent Sirt1 inhibitor, shows much lower potency against Sirt2 and Sirt3, and has no effect on Sirt5, but data for other isoforms are lacking. The novel compounds **3** and **4** are potent Sirt2 inhibitors and show only weak effects on Sirt1, 3, 5, and 6 (see text).

Sirtuin isoforms contribute to various key aspects of metabolic regulation, disease pathologies, and aging [1, 21]. They are thus considered attractive therapeutic targets for diseases such as cancer and neuro-degenerative disorders [22, 23], which has spurred interest in the mechanisms of Sirtuin catalysis and regulation and in small-molecule regulators for *in vivo* studies and therapy [22]. Inhibition of Sirt1 was shown to sensitize cells for DNA-damaging cancer therapeutics [24], and inhibition of Sirt1 and Sirt2 can itself decrease tumor growth [25, 26]. A variety of Sirtuin activating and inhibiting small molecules has thus been described [22, 23]. However, most of these compounds show limited potency, and their isoform specificity is often low or has not been tested. The widely used inhibitor sirtinol (**1**; Figure 1), for example, has an IC_50_ of 38 μM against Sirt2 in an *in vitro* assay, shows only ~3-fold weaker potency against Sirt1, and no data have been reported for its effect on other isoforms [23, 27, 28]. For Sirt1, EX-527 (**2**; Figure 1) was described as potent inhibitor with an IC_50_ of ~0.1 μM, and about two orders of magnitude lower potency against Sirt2 and Sirt3 and no effect against Sirt5, whereas no data are available for Sirt4, 6, and 7 [29]. Several more Sirtuin inhibitors have been described, but most of them resemble sirtinol, with reported IC_50_ in the higher μM range, comparable potencies against several isoforms, and no data for other isoforms [23, 30].

Crystal structures of the catalytic cores of bacterial and yeast Sirtuins as well as of mammalian Sirt2, 3, 5, and 6 reveal a conserved overall structure [31]. They contain a large Rossmann fold domain and a small, structurally more variable Zn^2+^-binding domain. The substrates, NAD^+^ and the acetyllysine side chain, enter the active site from opposite sides of a cleft between these do- mains, and the acetyl group then appears to be transferred via a 1'-O-alkylamidate reaction inter-mediate [4]. For several Sirtuin inhibitors, the lack of pronounced isoform specificity might be due to their potential binding to the pocket for the NAD^+^ cosubstrate common to all Sirtuin isoforms. Sirtuins have different protein targets, however, even if they are colocalized, like Sirt3 and 5 in mitochondria [13]. Although they show no strict sequence specificity, Sirtuins display residue preferences around the deacetylation site [32-34], and the polypeptide binding pocket thus should enable isoforms-specific contacts for inhibition. A mechanism-based, peptide-derived inhibitor indeed showed an IC_50_ of 4 μM for Sirt1, and ~17-fold and >77-fold lower potency against Sirt2 and Sirt3, respectively [35], indicating the peptide binding pocket as a promising target site. Interaction details with this and other inhibitors remain to be resolved, however, as the only inhibitor complex structure (other than complexes with non-specific NAD^+^ analogues) is the Sirt5 complex with suramin, a non-specific Sirt1/2 inhibitor partially occupying the NAD^+^ and peptide binding pockets [36].

**Table 1:** NCS numbers for top hits from the docking runs against Sirt2, 3, 5, and 6

Hit no.	Sirt2	Sirt3	Sirt5	Sirt6
**1**	23128 (CSC6^a^)	63875 (CSC15)	95609 (n.t.^b^)	51535 (CSC14)
**2**	115448 (CSC27)	234766 (CSC34)	371878 (n.t.)	299137 (CSC36)
**3**	13987 (n.t.)	13728 (CSC5)	282058 (CSC35)	74702 (CSC17)
**4**	11241 (CSC1)	94820 (CSC20)	74702 (CSC17)	13987 (n.t.)
**5**	74702 (CSC17)	13726 (CSC4)	105550 (CSC25)	94820 (CSC20)
**6**	99550 (CSC24)	99543 (CSC23)	122140 (CSC29)	12363 (CSC3)
**7**	128609 (CSC31)	95609 (n.t.)	135371 (CSC33)	79050 (CSC18)
**8**	299137 (CSC36)	343227 (n.t.)	13987 (n.t.)	13728 (CSC5)
**9**	90318 (CSC19)	23128 (CSC6)	95090 (CSC21)	135371 (CSC33)
**10**	94820 (CSC20)	26645 (CSC8)	125252 (CSC30)	23128 (CSC6)
**11**	12339 (CSC2)	79050 (CSC18)	300545 (CSC37)	63875 (CSC15)
**12**	111326 (CSC26)	99550 (CSC24)	13728 (CSC5)	37245 (CSC12)
**13**	402959 (CSC40)	132230 (CSC32)	23128 (CSC6)	371878 (n.t.)
**14**	35949(CSC11)	35049 (CSC9)	36806 (n.t.)	13726 (CSC4)
**15**	234766 (CSC34)	35489 (CSC10)	128609 (CSC31)	23217 (CSC7)
**16**	309883 (CSC38)	74702 (CSC17)	119886 (CSC28)	39863 (CSC13)
**17**	371878 (n.t.)	99515 (CSC22)	351123 (CSC39)	72254 (CSC16)
^a^ For chemical information on CSC compounds see Table 2.				
^b^ not tested				

Despite of the limited structural information for Sirtuin/inhibitor complexes, more and more structures of different Sirtuin isoforms reveal their subtle differences. Here, we describe a structure-based approach for identifying novel, isoform-specific inhibitors for human Sirtuins. Using crystal structures of human Sirt2, 3, 5, and 6, we identified potential ligands for the peptide binding grove through a docking screen with a small molecule library. Characterization of the docking hits in *in vitro* assays reveal two potent, Sirt2-specific compounds as well as a target site apparently enabling isoform specificity and additional compound scaffolds for further development, demonstrating the power of this approach for the development of specific Sirtuin inhibitors.

## RESULTS

### Identification of candidate compounds through a docking screen

Despite the physiological and therapeutic importance of Sirtuins [22], there is a paucity of potent, isoform-specific inhibitors [23, 30]. For the identification of novel Sirtuin inhibitor classes, we used the available crystal structures of human Sirt2 (PDB entry 1J8F)[37], Sirt3 (3GLS)[38], Sirt5 (2NYR)[36], and Sirt6 (3K35)[39] in docking screens. To avoid compounds blocking the NAD^+^ binding site, which is similar in different Sirtuin isoforms, we used complexes of the four Sirtuins with the NAD^+^-fragment ADP-ribose as receptor structures. Complexes were generated by transferring ADP-ribose from the experimental Sirt6/ADP-ribose structure to the other structures based on a superposition of the enzymes. The vacant space in the docking target site, defined as a cube centered around C1' of the ADP-ribose, thus corresponds to the pocket for recognition of the isoform-specific protein substrates and thus should allow isoforms-specific contacts.

For identifying potential ligands, we then docked the 1990 structurally diverse compounds of the National Cancer Institute (NCI) diversity set (http://www.dtp.nci.nih.gov) to the target sites of the four Sirtuins/NAD^+^ complexes. Despite some overlap between the hit lists, most of the compounds on top of the list for each isoform differed from the other isoforms (Table 1, 2; Supplementary Table 1). This result suggests that the chosen receptor sites indeed posses isoforms-specific structural features pronounced enough to be distinguishable for a docking approach, and thus for the identification of isoform-discriminating ligands.

### In vitro testing of docking hits reveals non-specific, semi-specific, and Sirt2-specific inhibitors

**Figure 2:**
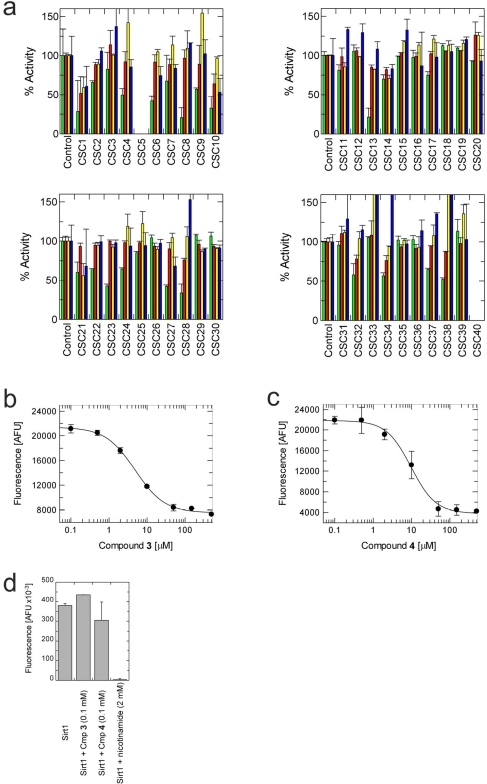
Effects of docking hits on the in vitro activity of Sirt2, 3, 5 and 6 (**a**) Shown is the relative activity compared to a control containing DMSO, which was used as solvent for all compounds. Green bars show Sirt2 activity, red Sirt3, yellow Sirt5, and blue Sirt6. CSC5 and 40 were not compatible with the assay used. (**b**) Concentration-dependent inhibition of Sirt2 activity by compound **3**. (**c**) Concentration-dependent inhibition of Sirt2 activity by compound **4**. (**d**) Effects of 100 mM compound **3** or **4**, respectively, on the in vitro activity of Sirt1. Inhibition of Sirt1 by 2 mM nicotinamide is shown as a control.

To evaluate the inhibitory potency and specificity of the identified potential ligands, we selected the top ten to seventeen hits of all four docking runs, resulting in 40 different compounds after removing NCS compounds 13987, 95609, 371878, and 343227, which we previously found to be incompatible with our assay. These compounds were then tested in *in vitro* activity assays for their effects against each of the four Sirtuin isoforms, Sirt2, 3, 5, and 6 (Figure 2a, Table 2). The experiments showed that two compounds were incompatible with the assay, and yielded varying types of results for the remaining 38 compounds. In total, 20 compounds showed significant inhibitory effects (>25% loss of activity) on one or several isoforms at the 100 μM compound concentration used, whereas 18 compounds had no such effect (Table 3). The high hit rate of 53% for the general ability to inhibit Sirtuins is comparable to hit rates in other library docking screens [40, 41] and indicates a successful enrichment of true ligands on top of the hit lists. Of the 20 inhibitory compounds, six (30% of the inhibitory ones) inhibited more than one isoform; five of the six (25%) were “semi-specific”, i.e. inhibited more than one but not all isoforms tested, whereas one compound (CSC1) behaved as a “broad-band” Sirtuin inhibitor that inhibited all isoforms. Surprisingly, all 14 compounds with a significant inhibitory effect against only one isoform were selective for Sirt2, yielding a panel of potential lead structures for the development of Sirt2-specific inhibitors. For Sirt3, 5, and 6, for which no specific modulators have yet been described, the five “semi-specific” inhibitors could still be interesting as leads (see below), especially the three compounds that just affect two isoforms.

### The specific compounds 3 and 4 show high potency against Sirt2

Of the tested compounds, 14 showed specificity for Sirt2 based on our selection criterion of >25% inhibition. From this panel of potential leads for the development of Sirt2-specific inhibitors, we picked the two compounds that showed the largest effects in the screen for further characterization. The compounds 3-hydroxyestra-1,3,5[10]-trien-17-yl 3-phenylpropanoate (CSC8, **3**; Figure 1) and 1,11a,13a-trimethyl-8-phenyl-2,3,3a,3b,4,5,5a,6,11,11a,11b,12,13,13a-tetradeca-hydro-1H-cyclopenta[5,6]naphtho[1,2-g]quinazolin-1-ol (CSC13, **4**; Figure 1) inhibited Sirt2 to ~80% in the screen, but had little or no effect on the other Sirtuin isoforms tested. A further characterization in dose-response experiments at a substrate peptide concentration of 100 μM revealed IC_50_ values of 4.8 ± 0.5 μM for **3** and 9.7 ± 1.5 μM for **4** (Figure 2b,c). We also tested the effects of **3** and **4**, respectively, on the *in vitro* activity of Sirt1 (Figure 2d). Both compounds had only a weak inhibitory effect at a concentration of 100 μM. Thus, both compounds are potent Sirt2 inhibitors with high isoform specificity.

It is noteworthy that **3** and **4** share considerable portions of their scaffolds (Figure 1). They both feature a steroid moiety, but modified at opposite ends; **3** is an estradiol with a bulky substituent at the 17-hydroxyl group, whereas **4** contains a steroid scaffold with a 2-benzyl pyrimidine fused to the A ring. Thus, the steroid scaffold is an attractive lead structure for the development at least of Sirt2-specific compounds, and even larger modifica-tions are possible, which should enable to avoid signify-cant side activity against steroid receptors (see below).

### Proposed binding orientation and molecular determinants for specificity

**Figure 3:**
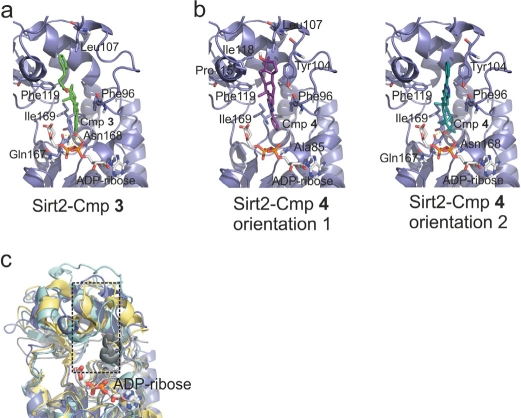
Models for the complexes between Sirt2/ADP-ribose and compounds 3 and 4, respectively (**a**) Docking model for the complex between the modeled Sirt2/ADP-ribose complex and **3**. Residues forming the binding pocket proposed to be occupied by the compound are shown in stick presentation and labeled. (**b**) Docking models for the complex between the modeled Sirt2/ADP-ribose complex and **4**. Two orientations representing poses about equally favored by the docking program are shown. Residues suggested to be involved in binding interactions are shown as sticks and labeled. (**c**) Overlay of the four Sirtuins studied here. Sirt2 is colored blue, Sirt3 yellow, Sirt5 cyan, and Sirt6 grey. Only the ADP-ribose of the Sirt2 complex is shown (sticks). The pocket suggested to bind **3** and **4** is indicated by a dotted box.

In order to gain insights into potential molecular determinants of compound specificity and starting points for compound variations in future development efforts, we analyzed the docking positions and orientations of **3** and **4** and differences in the respective binding sites between the Sirtuin isoforms (Figure 3a,b,c). In the model of the Sirt2 complex with **3** (Figure 3a), hydrophobic interactions are observed between the estradiol ring system and several side chains (Phe96, Leu107, Phe119 and Ile169) of a large, hydrophobic cavity (formed by the interacting residues and Ala85, Tyr104, and Ile118) extending into the small Sirtuin Zn^2+^-domain. In addition, the hydroxyl-group of **3** forms hydrogen bonds to the side chain of Asn168, the backbone of Gln167, and the α-phosphate-group of ADP-ribose.

For the Sirt2 complex with **4** (Figure 3b), two orientations were predicted with similar frequencies and binding energies. Orientation 1 shows only hydrophobic interactions between the sterol ring system and the hydrophobic pocket around Phe119 described above, whereas orientation 2 covers the same site, allowing less optimized hydrophobic interactions but in addition hydrogen bridges to Asn168 and Gln167 as observed in the model of the Sirt2 complex with **3**.

A comparison of the corresponding pockets in the four structurally characterized Sirtuin isoforms reveals that Sirt3, 5, and 6 have narrower pockets than Sirt2 (Figure 3c). In Sirt3, the hydrophobic residues are moved toward each other, closing the binding pocket compared to Sirt2. In Sirt5 and 6, there are bulkier residues (in both cases two Trp) lining the hydrophobic pocket, again making it smaller, and the protein backbone is partly moved into the pocket either from top-left (Sirt5) or bottom-right (Sirt6). These differences likely contribute to the observation in our screen, as well as from analysis of available compounds, that specific inhibition seems easier to achieve for Sirt2 than for Sirt3, 5, and 6. It suggests that for specific inhibition of the latter isoforms, smaller scaffolds should be favored (see below). For improvement of Sirt2 inhibitors, the docking model suggests that the hydrophobic substituent at position 17 of the sterol scaffold of **3** should be further extended. Such additional groups should contain polar functions, which could interact with polar protein groups or solvent at the pocket entrance and which would improve the solubility and possibly other properties of this compound.

2-hydroxy-2-((3-oxoestr-4-en-17-yl)oxy)-1H-indene-1,3(2H)-dione (CSC16, **5**) is also a steroid-based compound (Table 2), but despite of its similarity, especially to **3**, it had no effect on any of the tested Sirtuin enzymes. According to the structures and docking models, the binding pocket is too narrow for the compound in Sirt3 and 5, so that it was docked with low binding energy to the surface of the respective Sirtuin. In Sirt2 and 6, **5** could be docked in the hydrophobic pocket, but in both cases in a disadvantageous position, with carbonyl- and hydroxyl-oxygens of the compound near hydrophobic patches.

### Semi-specific compounds as inhibitor scaffolds and as lead compounds

**Figure 4:**
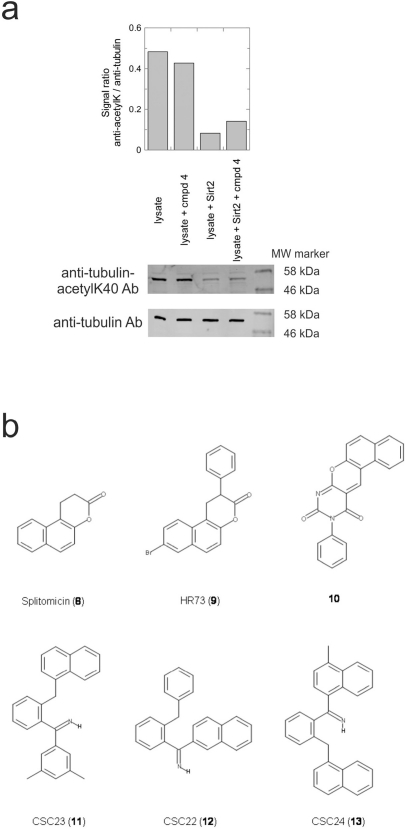
Effects of compounds 3 and 4 on Sirt2-dependent α-tubulin deacetylation and chemical structures of published Sirtuin inhibitors and compounds analyzed here (**a**) Lysates of HEK cells were incubated with Sirt2 in presence and absence of 100 μM compound **4** and then probed with anti-tubulin-acetylK40 antibody and anti-tubulin antibody, respectively. The bars show the signal ratios, indicating that the tubulin-deacetylating activity of Sirt2 is lowered by the addition of compound **4**. (**b**) Splitomicin (**8**) is an established inhibitor for yeast Sir2, and HR73 (**9**) and the tetracyclic pyrimidinedione **10** for mammalian Sirt1. **11**, **12**, and **13** are identified here as Sirt2-specific inhibitors, which form a series of structurally related compounds yet show varying inhibition potencies.

It is noticeable that for all compounds of our screen with an inhibitory effect, Sirt2 is either the only isoform affected or belongs to the group of isoforms that is inhibited. Most likely, this result is due to the larger hydrophobic binding pocket identified from analysis of the docking models (see above). However, compounds that inhibit Sirt2 and another isoform might still be developable into compounds specific for the other isoforms. The very large, hydrophobic compounds CSC1, CSC6, CSC14, and CSC27 (Table 2) likely cover non-specifically many large cavities and are unlikely to yield good pharmacological compounds, but CSC10 (**6**) and CSC21 (**7**; Table 2) appear promising. Due to its size, howeer, the essential parts of **7** should be determined in a structure-activity study before further development. **6**, in contrast, resembles compounds such as splitomicin (**8**), HR73 (**9**), and **10** (Figure 4), which were reported as micromolar inhibitors for a Sirtuin (**8** for yeast Sir2 [42], **9** and **10** for human Sirt1 [26, 43]), but which were not tested against most mammalian isoforms. No structural data are available for Sirtuin complexes with any of these compounds, but our structure comparison (see above) suggests that smaller scaffolds might enable these compounds to exploit the site where **3** was docked, which appears not to be accessible for bulkier compounds in Sirt3, 5, and 6. These scaffolds should thus be considered for the development of the first inhibitors specific for these Sirtuin isoforms. Considering that **6** already shows different potencies against the isoforms tested here (Figure 2), its phenanthrene and isochinoline moieties as well as similar groups should be further evaluated for specific Sirtuin inhibition.

### Activity of the novel Sirt2 inhibitors in a physiological system

Our novel compounds **3** and **4** are potent and specific Sirt2 inhibitors *in vitro*, and we next tested the effect of **4** (compound **3** is likely to be sensitive to esterase activity in lysates) on the Sirt2-dependent deacetylation of an *in vivo* substrate in a physiological environment. The acetylation levels of the Sirt2-deacetylation site Lys40 in α-tubulin [12] were analyzed in HEK cell lysates by using an acetyl-Lys40 specific antibody (Figure 4a). Adding Sirt2 strongly decreased the level of tubulin Lys40 acetylation in the lysate when no inhibitor was present, and the deacetylation effect was decreased in presence of 100 μM compound **4**. Thus, compound **4** is an inhibitor for the physiological α-tubulin deacetylation activity of Sirt2.

We further examined the likely suitability of **3** and **4** for *in vivo*use by applying analyses comparable to Lipinski's rule of five (using the molinspiration server at www.molinspiration.com). Both compounds appear generally suitable as pharmacological compounds, except for their lack of sufficient polar groups, leading to an unfavourable partitition coefficient. Compound **3** is further predicted to likely act as a ligand for nuclear receptors. In fact, the sterol ester **3** might be hydrolyzed in a physiological environment and thus generate a generic steroid receptor ligand. Compound **4**, instead, was not predicted to bind to nuclear receptors, most likely due to the larger deviation from the steroid scaffold. Testing on HEK cells revealed very high cytotoxicity for **3** (compound **4** showed a smaller effect but possibly due to partial precipitation under these conditions), but it remains to be clarified whether the corresponding mechanism is based on Sirt2 inhibition. However, **3** and **4** are potent and isoforms-specific Sirt2 inhibitors, and especially 4 appears promising for the development of side effect-free Sirt2 inhibitors, with adding suitable polar groups to this scaffold being a first and obvious optimization step.

## DISCUSSION

The roles of Sirtuins in central physiological processes and as drug targets have led to great demand for specific inhibitors for research and therapy [22, 23]. Available compounds often feature limited or unknown specificities and mostly high micromolar potencies, and surprisingly little structural information is available for Sirtuin/inhibitor complexes, which could be used for rational improvement. However, we show here that the increasing number of Sirtuin isoform structures in non-inhibited state [31] allow the structure-based identification of novel, isoform-specific inhibitor classes. A previous docking study on Sirt2 [44], the only structurally characterized mammalian isoform at that time, yielded only two Sirt2 inhibitors with IC_50_ values below 100 μM (57 and 74 μM, respectively), and their isoform specificities were not evaluated. The lower hit rate and affinities than in our study might have several reasons. Tervo et al. used a structure as docking receptor that had undergone a molecular dynamics (MD) simulation, resulting in major deviations from the experimental structure, which might not well represent the major conformations of the protein. To take protein flexibility into account, using several structures - representing different conformers - simultaneously, or simulating flexible side-chains during docking are now often used approaches [45]. The first option is not yet possible for most Sirtuins due to the lack of such multiple structures, and it appears that ignoring receptor flexibility is in fact a viable strategy for identifying Sirtuin inhibitors, at least for Sirt2. To take side chain flexibility into account, however, might be an approach for identifying compounds specific for the other isoforms studied here, Sirt3, 5, and 6 [40]. A further difference to our approach was that several potential inhibitors were not considered for experimental testing due to missing features assumed to be important for inhibition, which might have removed potent compounds. Also, a different docking software (GOLD) and compound database were used (Maybridge). The Mabridge database, being much larger than the NCI diversity set used here, is unlikely to be a bottleneck, but it has been observed repeatedly that docking programs differ in their performance depending on the interaction type, e.g. small versus large and hydrophobicversus hydrophilic ligands [40]. Binding of the most potent inhibitors identified appears to be dominated by hydrophobic interactions with a large cavity, which was previously observed not to give best results with GOLD [40]. Finally, we used a receptor with partially occupied NAD^+^ binding site. Thereby, we tried to avoid to obtain compounds binding to the NAD^+^ binding site present in similar form in all Sirtuins, as well as other NAD-dependent enzymes, likely resulting in little specificity. This approach further takes into account that interactions with the bound NAD^+^ can contribute to inhibitor affinity. The docking models obtained indeed indicate that NAD^+^ forms part of the bottom of the pocket likely occupied by the inhibitors. However, instead of mainly occupying the peptide binding cleft, the compounds were preferentially docked perpendicular to this cleft, extending into a hydrophobic pocket in the Zn^2+^ domain. If this binding orientation is confirmed by structural studies, then fusing to compounds such as **6** smaller substituents exploiting the isoforms specific features of the peptide binding grooves might be an attractive approach for further improvement of inhibitor potency and specificity.

**Table 2:** Docking hits against Sirt2, Sirt3, Sirt5, and Sirt6 tested *in vitro*

cmp-no.	NCS no.	Chemical structure	Name
CSC1	11241	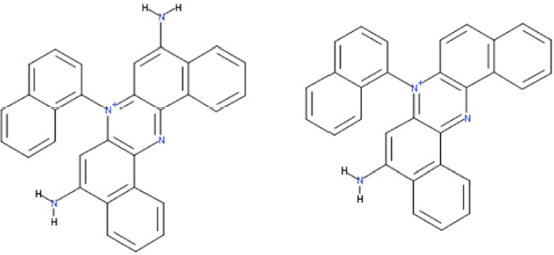	7-(1-naphthyl)-7^5^-dibenzo[a,j]phenazin-5-amine compound with 7-(1-naphthyl)-7^5^-dibenzo[a,j]phenazine-5,9-diamine (1:1), (Sudan Red)
CSC2	12339	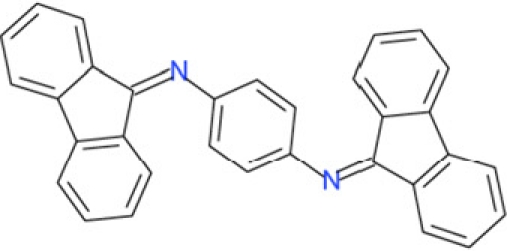	N1,N4-di(9H-fluoren-9-ylidene)-1,4-benzenediamine
CSC3	12363	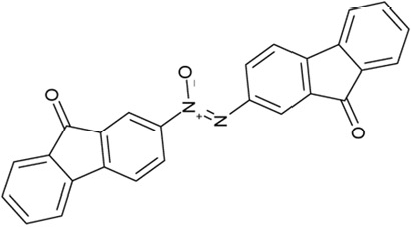	n.a.
CSC4	13726	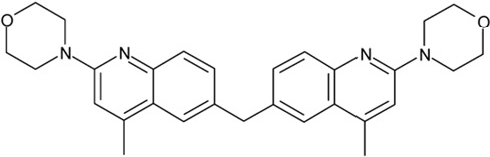	4-methyl-6-((4-methyl-2-(4-morpholinyl)-6-quinolinyl)methyl)-2-(4-morpholinyl)quinoline
CSC5	13728	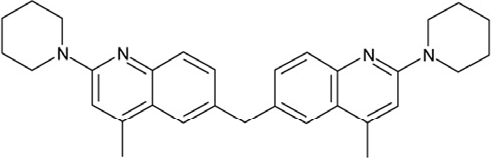	4-methyl-6-((4-methyl-2-(1-piperidinyl)-6-quinolinyl)methyl)-2-(1-piperidinyl)quinoline
CSC6	23128	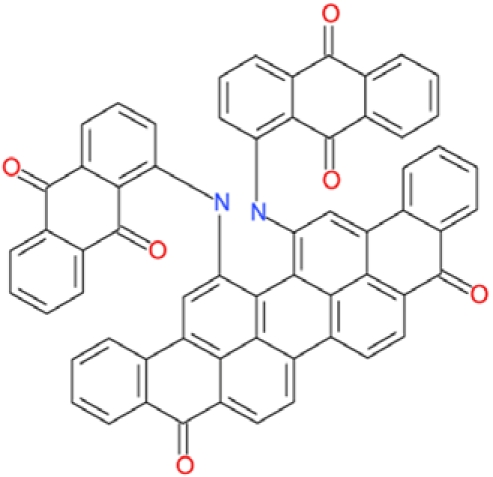	16,17-bis((9,10-dioxo-9,10-dihydro-1-anthracenyl)amino)anthra[9,1,2-cde]benzo[rst]pentaphene-5,10-dione
CSC7	23217	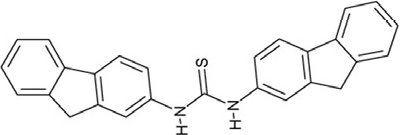	N,N'-di(9H-fluoren-2-yl)thiourea
CSC8	26645	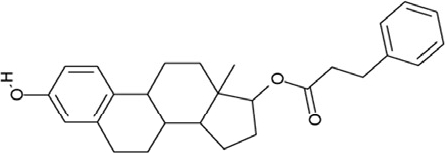	3-hydroxyestra-1,3,5(10)-trien-17-yl 3-phenylpropanoate
CSC9	35049	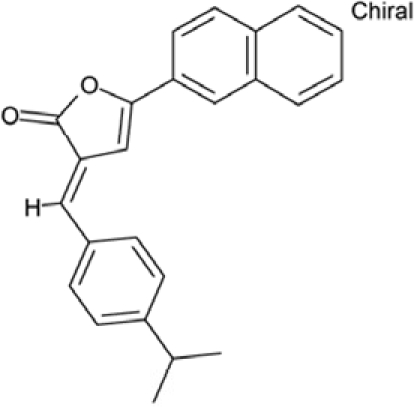	3-(4-isopropylbenzylidene)-5-(2-naphthyl)-2(3H)-furanone
CSC10	35489	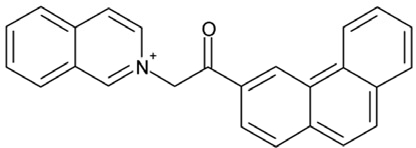	2-(2^5^-isoquinolin-2-yl)-1-(3-phenanthryl)ethanone
CSC11	35949	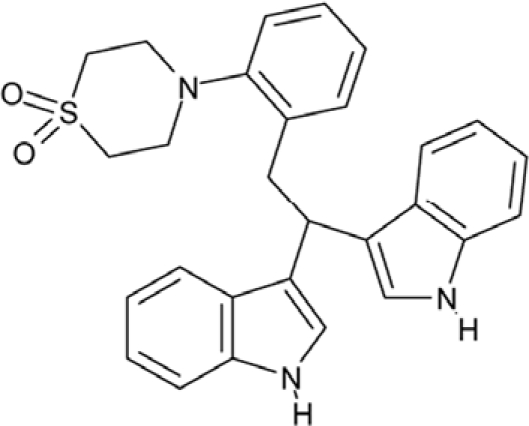	3-(2-(2-(1,1-dioxido-4-thiomorpholinyl)phenyl)-1-(1H-indol-3-yl)ethyl)-1H-indole
CSC12	37245	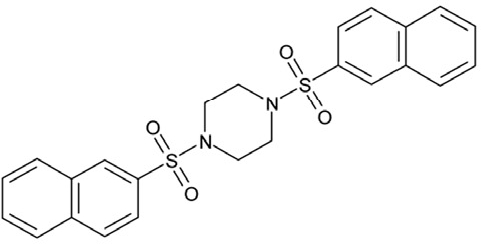	1,4-bis(2-naphthylsulfonyl)piperazine
CSC13	39863	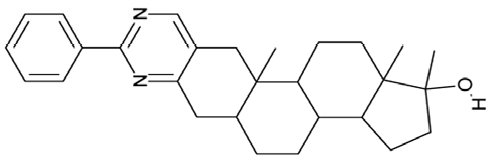	1,11a,13a-trimethyl-8-phenyl-2,3,3a,3b,4,5,5a,6,11,11a,11b,12,13,13a-tetradecahydro-1H-cyclopenta[5,6]naphtho[1,2-g]quinazolin-1-ol
CSC14	51535	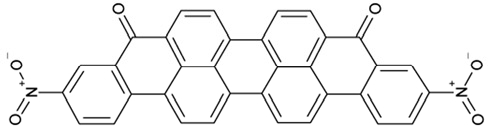	3,12bis(hydroxy(oxido)amino)anthra[9,1,2-cde]benzo[rst]pentaphene-5,10-dione, (Amanthrene Supra Black BBN)
CSC15	63875	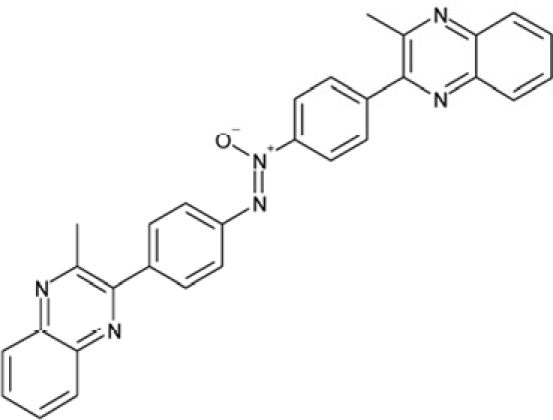	2-(4-(1-hydroxy-2-(4-(3-methyl-2-quinoxalinyl)phenyl)-1^5^-diazenyl)phenyl)-3-methylquinoxaline
CSC16	72254	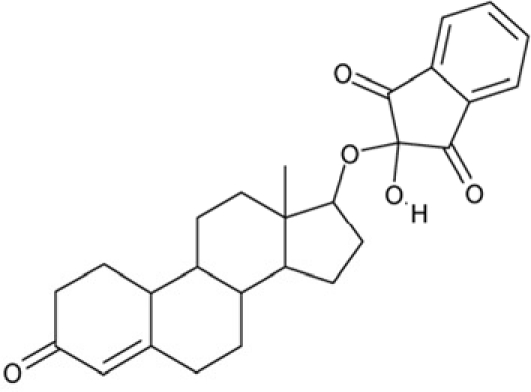	2-hydroxy-2-((3-oxoestr-4-en-17-yl)oxy)-1H-indene-1,3(2H)-dione
CSC17	74702	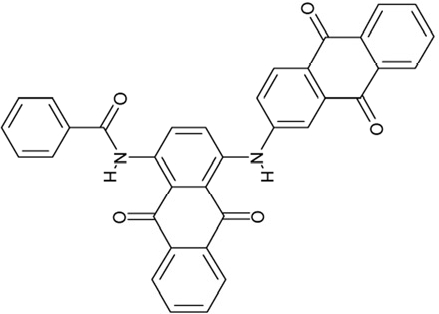	N-(4-((9,10-dioxo-9,10-dihydro-2-anthracenyl)amino)-9,10-dioxo-9,10-dihydro-1-anthracenyl)benzamide, (Indanthrene Corinth RK)
CSC18	79050	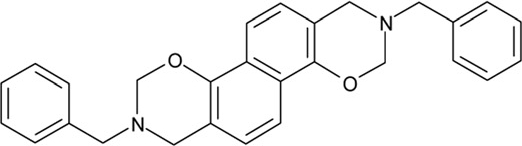	n.a.
CSC19	90318	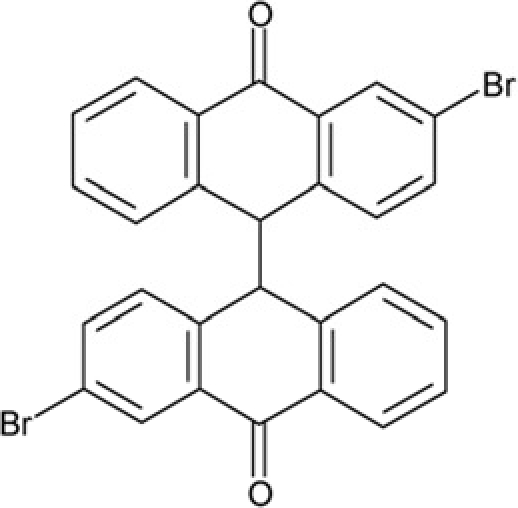	n.a.
CSC20	94820	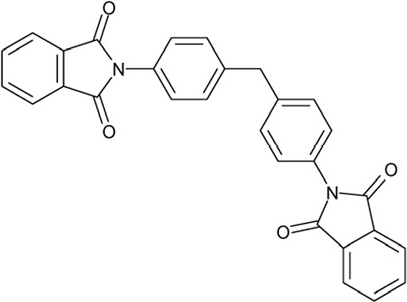	2-(4-(4-(1,3-dioxo-1,3-dihydro-2H-isoindol-2-yl)benzyl)phenyl)-1H-isoindole-1,3(2H)-dione
CSC21	95090	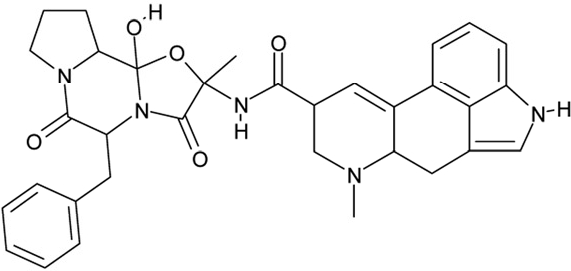	5'-benzyl-12'-hydroxy-2'-methyl-3',6',18-trioxoergotaman, (Ergotamine)
CSC22	99515	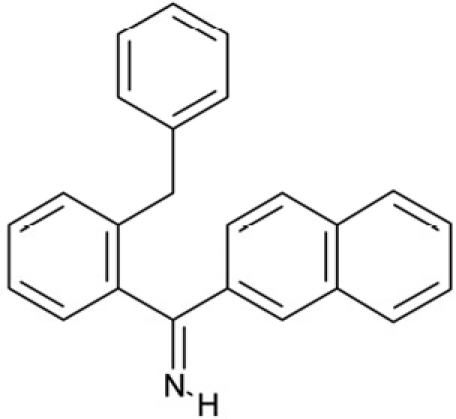	(2-benzylphenyl)(2-naphthyl)methanimine
CSC23	99543	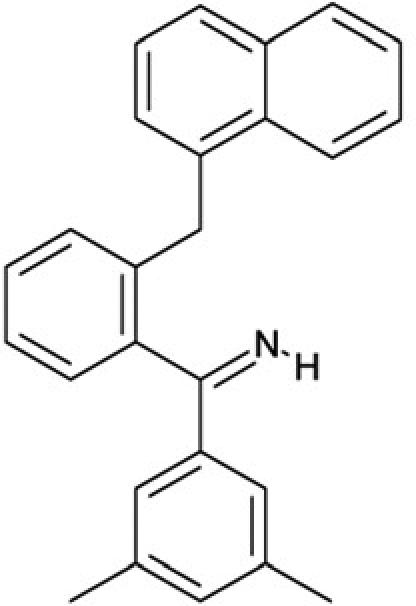	(3,5-dimethylphenyl)(2-(1-naphthylmethyl)phenyl)methanimine
CSC24	99550	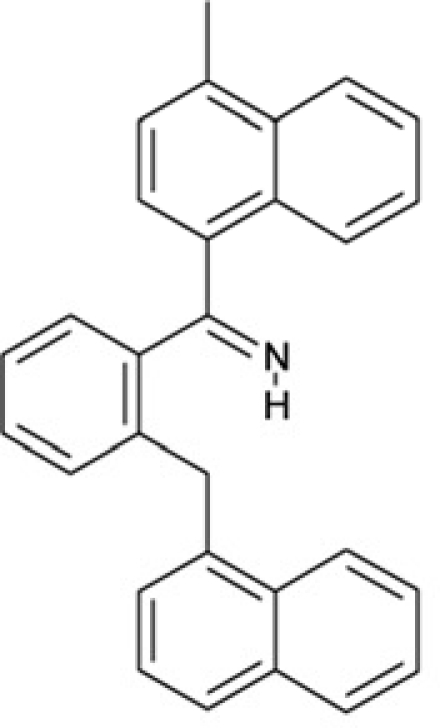	(4-methyl-1-naphthyl)(2-(1-naphthylmethyl)phenyl)methanimine
CSC25	105550	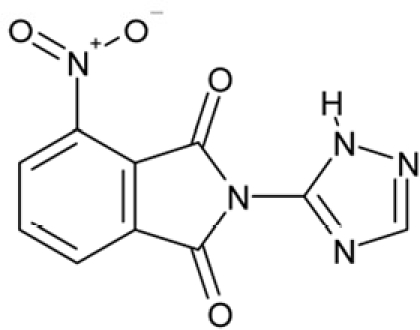	4-(hydroxy(oxido)amino)-2-(1H-1,2,4-triazol-5-yl)-1H-isoindole-1,3(2H)-dione
CSC26	111326	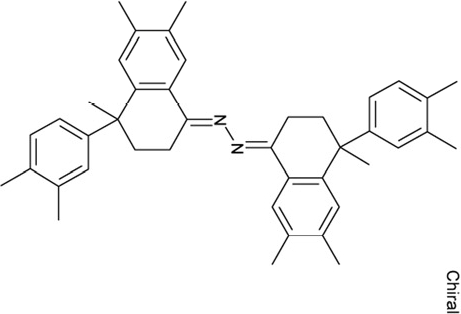	4-(3,4-dimethylphenyl)-4,6,7-trimethyl-3,4-dihydro-1(2H)-naphthalenone (4-(3,4-dimethylphenyl)-4,6,7-trimethyl-3,4-dihydro-1(2H)-naphthalenylidene)hydrazone
CSC27	115448	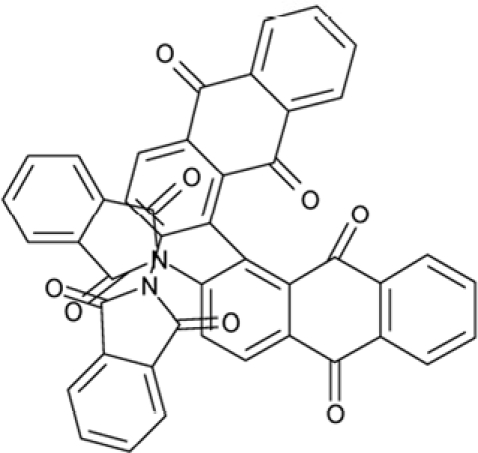	n.a.
CSC28	119886	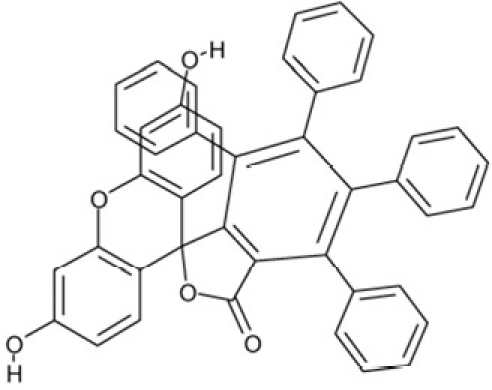	n.a.
CSC29	122140	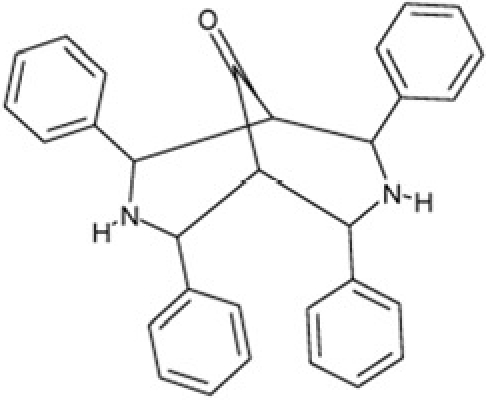	2,4,6,8-tetraphenyl-3,7-diazabicyclo[3.3.1]nonan-9-one
CSC30	125252	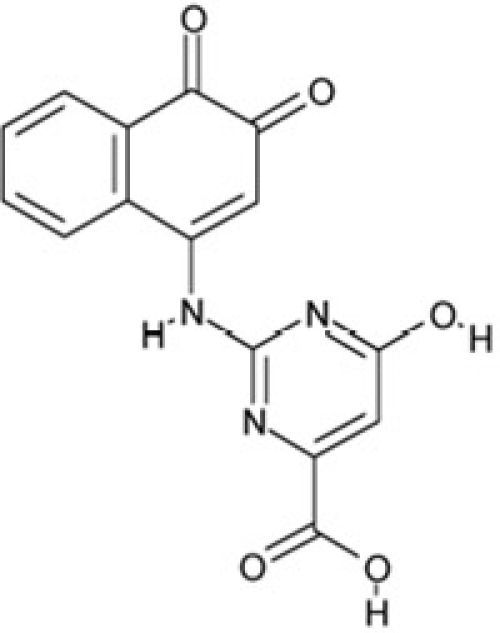	2-((3,4-dioxo-3,4-dihydro-1-naphthalenyl)amino)-6-hydroxy-4-pyrimidinecarboxylic acid
CSC31	128609	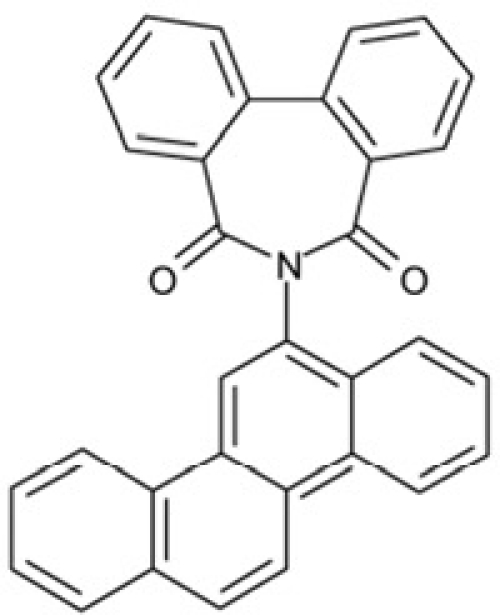	6-(6-chrysenyl)-5H-dibenzo[c,e]azepine-5,7(6H)-dione
CSC32	132230	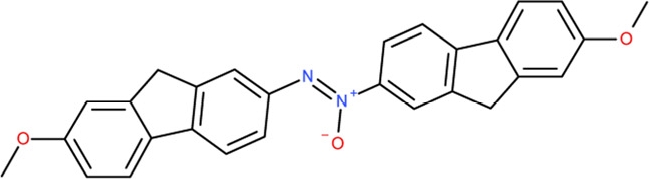	1-hydroxy-1,2-bis(7-methoxy-9H-fluoren-2-yl)-1^5^-diazene
CSC33	135371	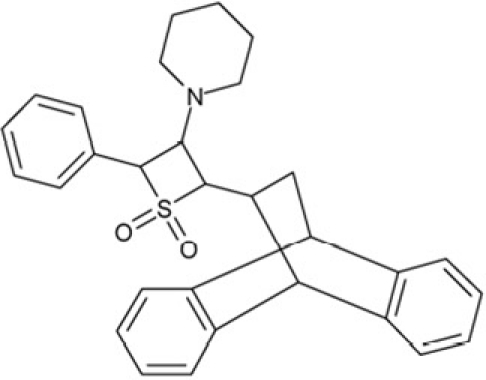	n.a.
CSC34	234766	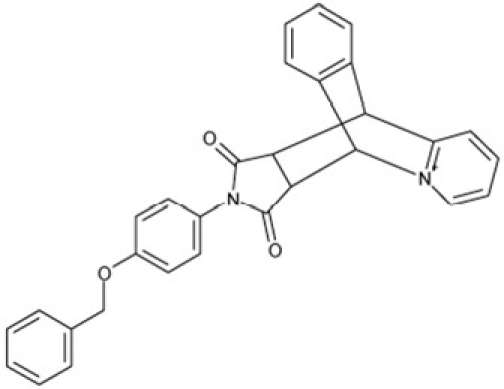	n.a.
CSC35	282058	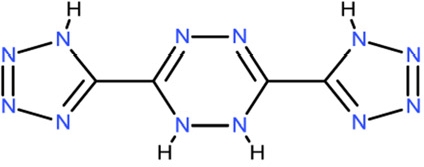	3,6-di(1H-tetraazol-5-yl)-1,2-dihydro-1,2,4,5-tetraazine
CSC36	299137	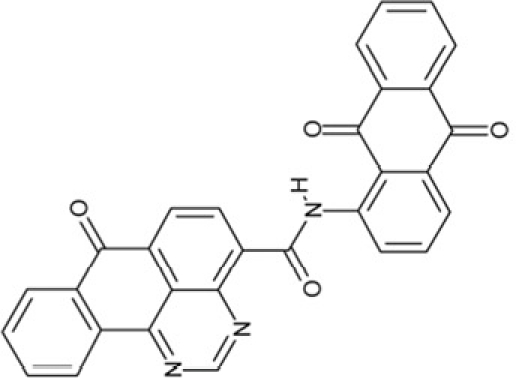	N-(9,10-dioxo-9,10-dihydro-1-anthracenyl)-7-oxo-7H-benzo[e]perimidine-4-carboxamide, (Pigment Yellow 108)
CSC37	300545	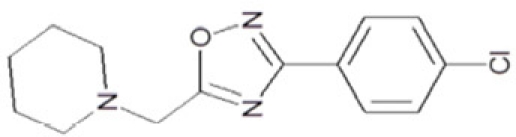	1-((3-(4-chlorophenyl)-1,2,4-oxadiazol-5-yl)methyl)piperidine
CSC38	309883	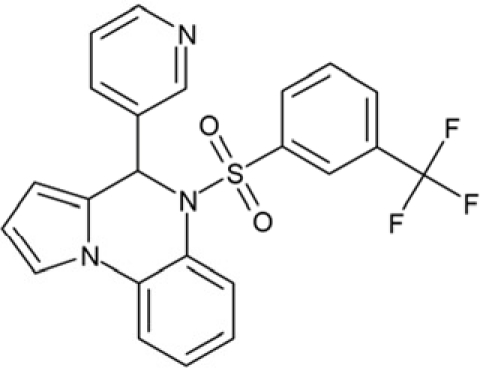	4-(3-pyridinyl)-5-((3-(trifluoromethyl)phenyl)sulfonyl)-4,5-dihydropyrrolo[1,2-a]quinoxaline
CSC39	351123	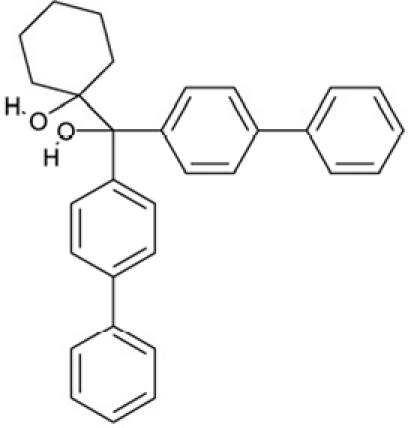	1-(di[1,1'-biphenyl]-4-yl(hydroxy)methyl)cyclohexanol
CSC40	402959	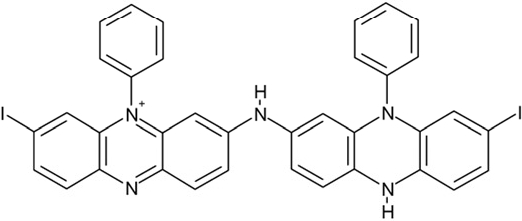	8-iodo-N-(8-iodo-10-phenyl-5,10-dihydro-2-phenazinyl)-10-phenyl-10^5^-phenazin-2-amine

Even though the NCI diversity set is supposed to represent a varying scaffold repertoire, it contains a number of series of structurally related compounds.

Interestingly, a series of related combinations of benzyl and naphtyl systems connected through a methylen and a methanimine bridge was identified in our screen (CSC22-24, Figure 4), and showed differences in the effects on the four Sirtuins tested (Figure 2a). All compounds inhibited only Sirt2, but CSC23 (**11**) had a larger effect than CSC22 (**12**) or CSC24 (**13**). They show a certain similarity to **1** (Sirtinol [28]; Figure 1) and the closely related Salermide [46], established Sirtuin inhibitors with some preference for Sirt2 over Sirt1 [23]. The observed differences suggest that further evaluating such combinations of three linked ring systems might have potential for further development of specific and potent inhibitors. Most promising for Sirt2 inhibition, however, appear the steroid-based compounds **3** and **4**, which should be further developed to overcome some disadvantages, such as limited polarity and solubility. Further, **3** should be sensitive to cellular esterase activities and is likely to act as a ligand for nuclear receptors, whereas **4** shows larger deviation from the steroid scaffold and thus might be the more promising lead for the development of side effect-free Sirtuin inhibitors. Several known Sirtuin inhibitors also comprise polycylic scaffolds, such as the Sirt1-over- Sirt2-selective pyrimidinedione **6**[26] and Splitomicin (**7**; [42]), which slightly resemble the A-C ring system of steroids and thus might exploit the same binding site. Attractive approaches for further development of these known and our novel inhibitors would be their rational modification, but the lack of structural information for these and most other Sirtuin inhibitors hinders such approaches. The putative binding pocket identified here, however, might already allow identifying improved Sirtuin inhibitors by screening of larger virtual libraries with more drug-like compounds, even before the anxiously awaited structural information on Sirtuin inhibitor complexes become available.

Improved compounds, derived from our Sirt2 inhibitors or identified through docking more drug-like compounds to their putative binding site, would be helpful for functional studies and promising for therapy. Acetylation of tubulin, a known Sirt2 deacetylation substrate [12], increases the stability of microtubule, and a reduction of tubulin acetylation in neurons has been reported for individuals with Alzheimer's disease [47]. Sirt2 inhibition could thus be an approach for therapy of this and other neurodegenerative diseases, such as Parkinson's disease, where Sirt2 inhibition was shown to prevent death of dopaminergic cells [48]. Sirt2 further contributes to regulation of cell cycle progression [49], and although details of its function seem to vary in different cancers, inhibition of Sirt2 seems to be a viable approach for destroying tumor cells [25, 26]. The other isoforms studied here might also be interesting drug targets, such as the mitochondrial forms Sirt3 and 5 for metabolic and aging-related diseases due to their role in regulating metabolic enzymes and stress-responses [21, 50]. Our data indicate that compounds with smaller ring systems, such as **6**, might better allow exploiting the tighter pockets of these isoforms corresponding to the predicted binding site for **3** and **4**. The binding site, however, is so far speculative, and the tighter conformation crystallized might just be one of several in solution, so that more information, such as Sirtuin/inhibitor complex structures or extensive structure-activity-relationship studies will be necessary for clear conclusions.

**Table 3:** Effects of the highest docking hits on the activity of Sirt2, 3, 5, and 6

Compound (NCS)	Sirt2	Sirt3	Sirt5	Sirt6
CSC1 (11241)	inhibition^a^	inhibition	inhibition	inhibition
CSC2 (12339)	inhibition	---	---	---
CSC3 (12363)	---	---	---	---
CSC4 (13726)	inhibition	---	---	---
CSC5 (13728)	n.c.^b^	n.c.	n.c.	n.c.
CSC6 (23128)	inhibition	---	---	inhibition
CSC7 (23217)	inhibition	---	---	---
CSC8 (26645)	inhibition	---	---	---
CSC9 (35049)	inhibition	---	activation^c^	---
CSC10 (35489)	inhibition	inhibition	---	inhibition
CSC11 (35949)	---	---	---	---
CSC12 (37245)	---	---	---	---
CSC13 (39863)	inhibition	---	---	---
CSC14 (51535)	inhibition	---	inhibition	---
CSC15 (63875)	---	---	---	---
CSC16 (72254)	---	---	---	---
CSC17 (74702)	---	---	---	---
CSC18 (79050)	---	---	---	---
CSC19 (90318)	---	---	---	---
CSC20 (94820)	---	---	---	---
CSC21 (95090)	inhibition	---	inhibition	inhibition
CSC22 (99515)	inhibition	---	---	---
CSC23 (99543)	inhibition	---	---	---
CSC24 (99550)	inhibition	---	---	---
CSC25 (105550)	---	---	---	---
CSC26 (111326)	---	---	---	---
CSC27 (115448)	inhibition	---	---	inhibition
CSC28 (119886)	inhibition	---	---	activation
CSC29 (122140)	---	---	---	---
CSC30 (125252)	---	---	---	---
CSC31 (128609)	---	---	---	---
CSC32 (132230)	inhibition	---	---	---
CSC33 (135371)	---	---	activation	activation
CSC34 (234766)	inhibition	---	---	activation
CSC35 (282058)	---	---	---	---
CSC36 (299137)	---	---	---	---
CSC37 (300545)	inhibition	---	---	---
CSC38 (309883)	inhibition	---	activation	activation
CSC39 (351123)	---	---	---	---
CSC40 (402959)	n.c.	n.c.	n.c.	n.c.

## EXPERIMENTAL SECTION

### 

#### Target Preparation and virtual ligand screening

For the docking study the crystal structures of human Sirt2 (PDB entry 1J8F), Sirt3 (3GLS), Sirt5 (2NYR), and Sirt6 (3K35) were used. In all structures, water molecules and ligands were removed except for ADP-ribose from the Sirt6 crystal structure, which was modeled in all other structures in the NAD^+^-binding site corresponding to its position in Sirt6. Polar hydrogen atoms were then added to the complex, and Kollman charges and AutoDock 4 atom type assigned by using AutoDockTools (http://autodock.scripps.edu/resources/adt). The docking site was defined as a box of 60 Å x 58 Å x 58 Å and was centered around the C1' atom of the ADP-ribose. For the virtual screen, the 1990 compounds of the NCI diversity set (http://www.dtp.nci.nih.gov) representing different scaffolds were docked into the Sirtuin receptor sites with AutoDock Vina [51]. The ligands were used in a form with all hydrogens added, computed Gasteiger charges, and assigned AutoDock 4 atom types available from http://autodock.scripps.edu/resources/databases.

For each Sirtuin [2,3,5,6] the 10 compounds with the best simulated binding energies in the docking study were further used for activity assays. Figures of structural models were generated with Pymol (http://www.pymol.org/).

#### Chemicals

The most promising docking hits were obtained from the NCI (http://www.dtp.nci.nih.gov). All other chemicals were obtained from Sigma (Saint Louis, USA) if not stated differently.

#### Cloning, recombinant expression, and purification of human Sirt1, 2, 3, 5 and 6

Human gene fragment comprising Sirt2 residues 34-356, Sirt3 residues 114-399, Sirt5 residues 34-302, and Sirt6 residues 13-308 were PCR amplified from full length cDNA clones and cloned into pET151/D-TOPO (Invitrogen, Carlsbad, USA), resulting in constructs with N-terminal his-tag and a linker comprising a TEV protease cleavage site. The full-length human Sirt1 gene was PCR amplified from a cDNA clone and inserted into vector pET15b (Merck Biosciences, Darmstadt, Germany) resulting in a thrombin cleavable his-tag at the N-terminus.

Sirt3 was expressed in *E. coli* and purified via Ni-NTA and size exclusion chromatography as described elsewhere [18], and Sirt5 according to the protocol of Schuetz et al. [36]. Sirt1 was expressed and purified as described for Sirt3, except that after affinity purification the protein was passed through a Superdex200 gel filtration column (GE Healthcare, New Jersy, USA) equilibrated in Buffer A (25 mM HEPES, pH 7.5, 100 mM KCl, 2 mM DTT), followed by HiTrapQ (GE Healthcare, Waukesha, WI, USA) anion exchange chromatography. The protein was bound to the HiTrapQ column in buffer A and eluted in a gradient to buffer A supplemented with 400 mM KCl. Fractions were analyzed by SDS-PAGE, and the Sirt1 fractions were pooled and transferred into buffer A using a NAP column (GE Healthcare).

The Sirt2 construct was expressed in *E. coli* Rosetta2 cells (Merck) in LB/Ampicillin/Chloramphenicol medium. Cells were grown at 37 °C, and expression induced at an OD_600_ of 0.6-0.8 for 4 h with 0.5 mM IPTG. Harvested cells were resuspended in buffer A (50 mM Tris/HCl pH 7.8, 200 mM NaCl, 2 mM DTT), disrupted with a Sonifier, and cell debris removed by 40 min centrifugation at 4 °C, 18000 rpm in a HFA22.50 rotor. The supernatant was supplemented with 10 mM imidazole and incubated with Ni-NTA resin for 1 h at 4 °C. The resin was washed in a column with 10 volumes buffer B (50 mM Tris/HCl, pH 7.8, 500 mM NaCl, 2 mM DTT) and 10 volumes buffer A supplemented with 10 mM imidazol, and eluted with 50 mM Tris/HCl, pH 7.8, 20 mM NaCl, 60 mM imidazol, 2 mM DTT. The protein was concentrated in an amicon unit (Millipore, Billerica, USA) and digested over night at 4 °C with TEV-protease. The protease was removed from cleaved Sirt2 by incubation with Ni-NTA resin for 1 h at 4 °C. The protein was concentrated, 1:5 diluted with 50 mM Tris/HCl, pH 7.8, and applied to a HiTrap Q anion exchange column (GE Healthcare) equilibrated with 50 mM Tris/HCl, pH 7.8, 50 mM NaCl, 2 mM DTT and eluted with a gradient (20 column volumes) to 50 mM Tris/HCl, pH 7.8, 1 M NaCl, 2 mM DTT. Sirt2 was then applied to a Superdex200 gel filtration column (GE Healthcare) equilibrated in 20 mM Tris/HCl, pH 7.8, 150 mM NaCl, 2 mM DTT. The eluted protein was analyzed by SDS-PAGE, re-concentrated, and shock-frozen in liquid nitrogen for storage at -80 °C.

The Sirt6 construct was expressed in E. coli Rosetta2 cells and purified through NiNTA affinity chromatography, TEV cleavage, and ion exchange chromatography as described for Sirt2, except that cells were shaken over night at 20 °C after induction, pH7.0 was used for all buffers, and a HiTrap SP cation exchange column (GE Healthcare) was used for ion exchange chromatography.

#### Peptide deacetylation assay

The commercial Fluor-de-Lys (FdL) assay kits 1 and 2 (Biomol, Plymouth Meeting, USA) were used to test the deacetylase activity of the four Sirtuins. In both kits the substrate derived from p53 is composed of three amino acids, the acetyl lysine and a C-terminally attached fluorophor. For Sirt2 and 3, FdL-2 peptide (QPK(acetyl-K)) was used, and FdL-1 peptide (RHK(acetyl-K)) for Sirt5 and Sirt6. All tests were done with 0.1 mM of the respective FdL peptide and 0.5 mM NAD^+^ as substrates.

NCI compound stocks were prepared by dissolving them in DMSO at a concentration of 5 mM. The initial screens with all 40 compounds contained 5 μl Sirtuin solution (Sirt2/3 0.1 mg/ml stock concentration; Sirt5/6 1 mg/ml), 1 μl of compound stock (resulting in a 100 μM end concentration), 15 μl FdL substrate, and assay buffer (supplied in the assay kit) to a total volume of 50 μl. As control reactions, peptide with either the compound (without Sirtuin) or with Sirtuin and DMSO instead of compound were measured. After starting the reactions by adding the FdL substrate, assays were incubated for 30 min at 37 °C. Subsequently, 50 μl of a 10 mg/ml trypsin stock (“Developer solution”) were added and the reactions were incubated for 45 min at room temperature. Fluorescence values were measured in a fluorometric plate reader with excitation at 360 nm and read out at 485 nm. Values were then corrected with the control reaction (same compound, but without Sirtuin). For dose-response curves, concentrations of the compounds were varied (0.1, 0.5, 2, 10, 50, 150 and 500 μM final concentrations in the assay) by diluting stock solution in DMSO so that constant volumes were added, except for the highest concentration, where a higher final DMSO concentration had to be accepted.

All measurements shown are averages of duplicates (screen) or triplicates (dose-response curves), and representatives of at least two independent replications. Error bars indicate standard deviations.

#### Tubulin deacetylation assay

HEK 293T cells were grown in DMEM medium (PAN Biotech) supplemented with 10% fetal bovine serum (PAN Biotech) at 37°C and 5% CO_2_. Cells were harvested, washed with PBS, and resuspended and lysed using hypotonic buffer (10mM Tris-HCl, pH 8.0, 3mM KCl) supplemented with protease inhibitors cocktail. Cell debris was removed by centrifugation (10,000g, 10 minutes) at 4°C, and 50μl reaction were prepared with identical amounts of cell lysate and either 100 μM compound **4**, 1 μg Sirt2, or both. All samples further contained the same amount of DMSO (to account for the solvent of the stock of **4**) and 25 mM HEPES (pH 7.5; to compensate for the solvent of the NAD^+^ stock). 20 μg total protein was then loaded on a gel for blotting with the monoclonal anti-acetylated tubulin antibody (Sigma), and 10 μg total protein for the blot with anti-tubulin antibody (Epitomics, Burlingame, CA, USA). Proteins were blotted to polyvinyl-idenfluorid membrane, incubated with primary (2 h) and then secondary antibodies at room temperature(1h; anti-Mouse IR Dye 680LT and goat anti-Rabbit IR Dye 680LT (Li-Cor Biosciences, Lincoln, NE, USA), respectively), and the blots were scanned in the 700 nm channel using an Odyssey imaging system (Li-Cor).

## SUPPORTING INFORMATION

Supplementary Table 1 Hit position of the 40 tested compounds in the docking runs against Sirt2, 3, 5, and 6
