# Supplementary material for: Structure-based development of novel sirtuin inhibitors
Source: Aging (Albany NY). 2011 Sep 20;3(9):852–72. doi: 10.18632/aging.100388 (PMC3227451; doi:10.18632/aging.100388)
Supplement: Supplementary Table 1 [file aging-03-852-s001.doc]

| **Compound (NCS)** | **Sirt2** | **Sirt3** | **Sirt5** | **Sirt6** |
| --- | --- | --- | --- | --- |
| CSC1 (11241) | 4 | --- | --- | --- |
| CSC2 (12339) | 11 | --- | --- | --- |
| CSC3 (12363) | --- | --- | --- | 6 |
| CSC4 (13726) | --- | 5 | --- | --- |
| CSC5 (13728) | --- | 3 | --- | 8 |
| CSC6 (23128) | 1 | 9 | --- | 10 |
| CSC7 (23217) | --- | --- | --- | 15 |
| CSC8 (26645) | --- | 10 | --- | --- |
| CSC9 (35049) | --- | 14 | --- | --- |
| CSC10 (35489) | --- | 15 | --- | --- |
| CSC11 (35949) | 14 | --- | --- | --- |
| CSC12 (37245) | --- | --- | --- | 12 |
| CSC13 (39863) | --- | --- | --- | 16 |
| CSC14 (51535) | --- | --- | --- | 1 |
| CSC15 (63875) | --- | 1 | --- | --- |
| CSC16 (72254) | --- | --- | --- | 17 |
| CSC17 (74702) | 5 | --- | 4 | 3 |
| CSC18 (79050) | --- | --- | --- | 7 |
| CSC19 (90318) | 9 | --- | --- | --- |
| CSC20 (94820) | 10 | 4 | --- | 5 |
| CSC21 (95090) | --- | --- | 9 | --- |
| CSC22 (99515) | --- | 17 | --- | --- |
| CSC23 (99543) | --- | 6 | --- | --- |
| CSC24 (99550) | 6 | --- | --- | --- |
| CSC25 (105550) | --- | --- | 5 | --- |
| CSC26 (111326) | 12 | --- | --- | --- |
| CSC27 (115448) | 2 | --- | --- | --- |
| CSC28 (119886) | --- | --- | 16 | --- |
| CSC29 (122140) | --- | --- | 6 | --- |
| CSC30 (125252) | --- | --- | 10 | --- |
| CSC31 (128609) | 7 | --- | --- | --- |
| CSC32 (132230) | --- | 13 | --- | --- |
| CSC33 (135371) | --- | --- | 7 | 9 |
| CSC34 (234766) | --- | 2 | --- | --- |
| CSC35 (282058) | --- | --- | 3 | --- |
| CSC36 (299137) | 8 | --- | --- | 2 |
| CSC37 (300545) | --- | --- | 11 | --- |
| CSC38 (309883) | 16 | --- | --- | --- |
| CSC39 (351123) | --- | --- | 17 | --- |
| CSC40 (402959) | 13 | --- | --- | --- |
